# Mapping higher-order relations between brain structure and function with embedded vector representations of connectomes

**DOI:** 10.1038/s41467-018-04614-w

**Published:** 2018-06-05

**Authors:** Gideon Rosenthal, František Váša, Alessandra Griffa, Patric Hagmann, Enrico Amico, Joaquín Goñi, Galia Avidan, Olaf Sporns

**Affiliations:** 1 Department of Cognitive and Brain Sciences, Ben-Gurion University of the Negev, P.O.B. 653, 8410501 Beer-Sheva, Israel; 2 The Zlotowski Center for Neuroscience, Ben-Gurion University of the Negev, P.O.B. 653, 8410501 Beer-Sheva, Israel; 3 Brain Mapping Unit, Department of Psychiatry, University of Cambridge, Cambridge, CB2 0SZ UK; 4 Department of Radiology, Centre Hospitalier Universitaire Vaudois (CHUV) and University of Lausanne (UNIL), 1011 Lausanne, Switzerland; 5 Signal Processing Laboratory 5 (LTS5), École Polytechnique Fédérale de Lausanne (EPFL), 1015 Lausanne, Switzerland; 6 School of Industrial Engineering, Purdue University, West-Lafayette, 47907 IN USA; 7 Purdue Institute for Integrative Neuroscience, Purdue University, West-Lafayette, 47907 IN USA; 8 Weldon School of Biomedical Engineering, Purdue University, West-Lafayette, 47907 IN USA; 9 Department of Psychology, Ben-Gurion University of the Negev, P.O.B. 653, 8410501 Beer-Sheva, Israel; 10 Department of Psychological and Brain Sciences, Indiana University, Bloomington, IN 47405 USA

## Abstract

Connectomics generates comprehensive maps of brain networks, represented as nodes and their pairwise connections. The functional roles of nodes are defined by their direct and indirect connectivity with the rest of the network. However, the network context is not directly accessible at the level of individual nodes. Similar problems in language processing have been addressed with algorithms such as word2vec that create embeddings of words and their relations in a meaningful low-dimensional vector space. Here we apply this approach to create embedded vector representations of brain networks or connectome embeddings (CE). CE can characterize correspondence relations among brain regions, and can be used to infer links that are lacking from the original structural diffusion imaging, e.g., inter-hemispheric homotopic connections. Moreover, we construct predictive deep models of functional and structural connectivity, and simulate network-wide lesion effects using the face processing system as our application domain. We suggest that CE offers a novel approach to revealing relations between connectome structure and function.

## Abstract (web summary)

The function of a brain region is determined by the network it is embedded in. Here the authors implement the word2vec algorithm for connectomes generating a vector embedding of the connectivity structure for each node allowing inference about functional relationships between brain regions.

## Introduction

An organism’s nervous system is composed of specialized brain regions, each associated with distinctive processing capacities and responses. However, these regions do not work in isolation, and in fact, a region’s functional role is tightly linked to its anatomical connectivity and physiological interactions with other regions in the system. The totality of these connections and interactions can be summarized, and systematically analyzed by using concepts of network science. Networks comprise a set of elements and their dyadic (pairwise) connections which allow characterizing each element’s connection pattern. The connectome offers such a network description, summarizing an organism’s complete nervous system as a graph which represents the complete set of connections between pairs of neurons or brain regions^1^. ^4,^).

Despite recent advances in connectome mapping^2^, the resulting collections of dyadic relations do not, by themselves, fully represent and quantify higher-order relations among nodes within the network. At the level of the network map, each node is defined by a vector corresponding to its connections with all other nodes, arranged in a high-dimensional topological space. Such a dyadic description does not readily allow visualization, classification, prediction of missing edges and nodes, and understanding relations between different networks^3, 4^. While there are many descriptive graph measures that can capture local and global network features^5^, most of these measures are not designed to capture the shape of the topological space within which individual nodes of the network are embedded. Assessing distances among connectivity profiles and subsequent dimension reduction (e.g., through PCA or multidimensional scaling) can reveal pairwise similarities^6^ but this approach does not capture other relations such as homologies or higher-order regularities.

Outside of connectomics, another field focused on mapping relationships between elements is natural language processing, where words may be represented or embedded in a low-dimensional distributed vector space^7, 8^. This representation can facilitate higher-level natural language processing tasks by grouping similar words into a similar embedded representation. One recent set of models for learning vector representations of words is word2vec which encodes linguistic regularities and patterns. These regularities may be manipulated using linear operations. For example, the result of a vector calculation vec(“King”)—vec(“Man”) + vec(“Woman”) is closest to vec(“Queen”) than to any other word vector^7–9^. Importantly, word2vec algorithms have been recently generalized for representing networks instead of text. The analog for sentences in the network domain are streams of randomly generated walks in the network (for example Deepwalk, Node2vec, and Grarep;^3, 4, 10^). The resultant latent nodes representation captures neighborhood similarity and community membership in a continuous vector space with a relatively small number of dimensions^4^. These low-dimensional embeddings are useful for subsequent machine learning applications directed at uncovering structural relations and similarities.

Here we build upon these advances with an embedded representation of the human connectome (connectome embedding; CE). The aim is to capture the structural network-level relations between brain regions in a low-dimensional continuous vector space to allow inferences about their functional roles and relationships. We suggest that CE provides a general approach for modeling connectome data that has many potential applications, including development, individual differences and clinical/translational studies.

**Fig. 1:**
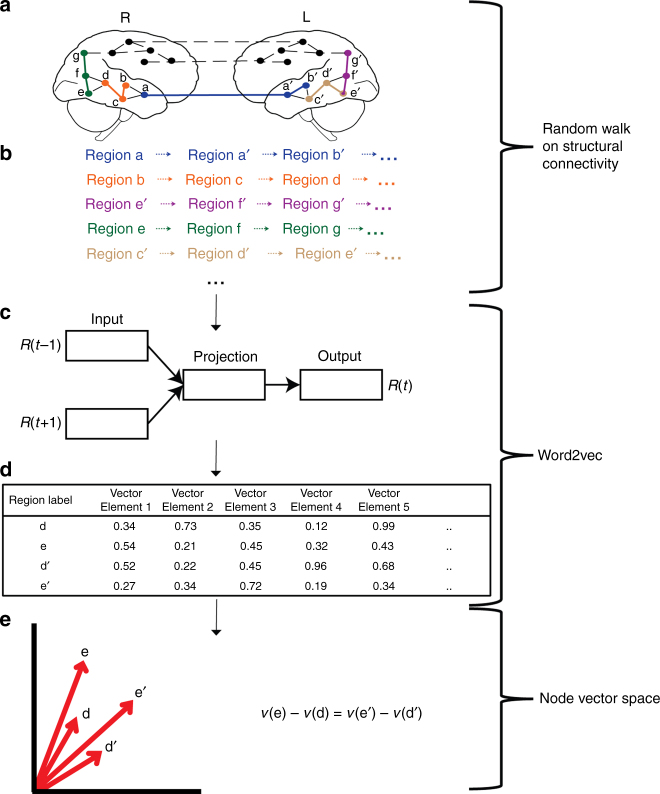
Connectome embeddings workflow. **a** The input to the connectome embeddings algorithm is a structural connectome, describing pairwise connectivity between brain regions (nodes). Letters denote unique, corresponding nodes in the right and left hemispheres of the brain (for example a and a’ represent homotopic regions). Dashed lines represent possible direct edges between regions according to the structural connectivity matrix, while full lines represent random walks [3]. **b** Random walks are performed on the network using the node2vec algorithm producing node sequences (note that we present sequences of 3 steps for demonstrational purposes, the sequence may be longer). **c** The sequence for each node is used as an input to a word2vec Continuous Bag of Words (CBOW) algorithm. Briefly, for each sequence, each node in turn is considered a target, *R*(*t*), which is predicted from the other nodes in the same sequence [*R*(*t* − 1), *R*(*t* + 1)..]. The goal is to maximize the conditional probability *p*(*R*(*t*)| *R*(*t* − 1), *R*(*t* + 1)..; *θ*) by estimating the parameters *θ* using a 2 layers neural network (Goldberg & Levy, 2014). **d** The obtained parameters *θ*, or vectors capture regularities and may be the basis for various subsequent tasks [7, 8] which forms a vector distributed representation of each node. **e** The direction of the produced vector representation of a node has a topological meaning. For example, the differences between homological nodes in opposing hemispheres are analogs (see results for details)

To test the utility of CE, first, a network embedding algorithm is used to embed a diffusion structural MRI connectome into a continuous vector representation, or CE (Fig. 1a-e). Next, we demonstrate that CE representations are neurobiologically meaningful and can be manipulated using linear operations. Then, we demonstrate that CE representations can predict functional connectivity from structural connectivity with high accuracy for both direct and indirect connections. Lastly, we use CEs to predict network-level functional effects of localized lesions in structural networks.

## Results

### Inter-hemispheric analogies test

To test whether CE vector representations are consonant with known attributes of brain topology/topography and can be interpreted and manipulated using linear operations, we formulated a brain specific benchmark, namely, an inter-hemispheric analogies test.

One of the basic organizational characteristics of the human brain is functional homotopy, i.e., symmetric inter-hemispheric correlations between bilaterally homologous brain regions^11–14^. Functional homotopy is supported by a high proportion of callosal fibers contributing to homotopic connectivity^14, 15^. Moreover, the structural and functional connectivity patterns of the two hemispheres exhibit high levels of cross-hemisphere similarity. Consequently, to design a benchmark for testing and tuning connectome embeddings, we postulated that the relation between each pair of regions in one hemisphere should be analogous to the same pairwise relation in the other hemisphere. We tested all possible inter-hemispheric analogies between all nodes for both node2vec and spectral embedded vectors. For each analogy, the cosine similarity^7–9^ which ranges between −1 and 1 and captures the cosine of the angle between two vectors, was computed between a linearly combined vector [vector(“Right Node A”)—vector (“Right Node B”)+vector (“Left Node B”)] and all of the nodes vector embeddings. This procedure produced a vector of cosine similarity distances which was then ranked in an ascending order. The rank of the expected vector (“Left Node A”) was logged for each analogy. We term this procedure the inter-hemispheric analogies test.

We benchmarked our results against a more standard spectral embedding algorithm which is an unsupervised method aimed at calculating low dimensional non-linear embeddings of the data using a decomposition of the graph Laplacian^16, 17^. One of the strong underlying assumptions of spectral embedding is that interconnected nodes should be embedded together in the vector space (homophily) and that these embeddings are useful for classification. This might not be the case for some networks and tasks, such as the current analogies task, which requires a representation of the structural role of each node (structural equivalence) or a mixture between homophily and structural equivalence^3, 18^.

For each hemispheric analogy (vectors’ linear combinations; see methods for details), the median rank was calculated across 500 node2vec iterations. For example, vec(“Left Amygdala”)—vec(“Left Fusiform Gyrus”) + vec(“Right Fusiform Gyrus”) should yield a vector that has the smallest distance to vec(“Right Amygdala”) compared with all other node vector embeddings (see Fig. 1e). If the calculated vector is indeed closest to vec(“Right Amygdala”), then the rank difference of the analogy would be 0; Hence we termed this node (in this example, the Right Amygdala) as the expected node. Note that higher ranking means that the expected node embedding was less similar to the calculated vector and consequently higher ranking reflects worse performance. Thus, if across all possible analogies, a high proportion of the expected nodes would have a low rank (a small distance from the linearly combined vector), then one could infer that the obtained vector representations encompass meaningful topological information.

**Fig. 2:**
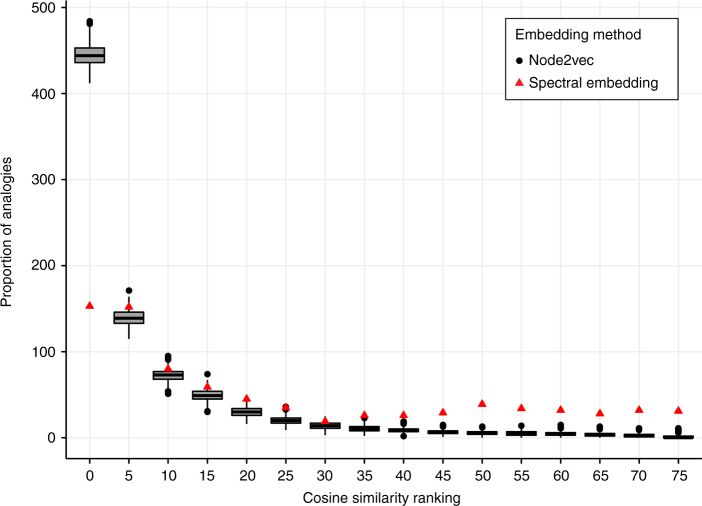
Performance of two node embedding algorithms on the inter-hemispheric analogies test. The inter-hemispheric analogies test evaluated the capacity of two node embeddings to infer the relation between each pair of nodes in one hemisphere, given the same pairwise relation in the other hemisphere. Predictions across all pairwise analogies were ranked, such that a lower rank corresponds to better performance. Across 500 iterations of the node2vec algorithm, the ranking of the expected nodes were binned into bins of 5. The boxplots represent the binning of the ranking across 500 node2vec permutations. The band inside the box represents the median, the lower and upper hinges correspond to the first and third quartiles (the 25th and 75th percentiles) and the whiskers represent 1.5 times the inter-quartile range (the distance between the first and third quartiles). The red triangles represent the binning of the spectral ranking. Importantly, the node2vec algorithm produced a higher proportion of expected nodes in the lowest rank bin (0–5 ranking). Note the relatively high proportion of spectral embedding analogies with high ranking which suggest worst performance in this task. This result demonstrates that node2vec vector embeddings successfully encompass functional homotopy information

Dataset 1: Across all hemispheric analogies, 54% of the expected nodes, 444 out of 820 (number of possible inter hemispheric analogies with 82 homologous nodes), were ranked as one of the top five nodes when using connectome embedding. In contrast, only 18.6%, 153 out of 820, of the expected nodes were ranked in the top five nodes using a conventional spectral embedding algorithm. The percentage of the top 5 ranked nodes significantly differed across these two embedding methods, *χ*^2^(1, *N* = 1640) = 223, *p* < 2.2e-16 (Fig. 2). Dataset 2: As was the case for Dataset 1, the difference between the connectome embedding and the spectral clustering analogies was significant. Across all hemispheric analogies, 30% of the expected nodes, 246 out of 820 (number of possible inter hemispheric analogies with 82 homologous nodes), were ranked as one of the top five nodes when using connectome embedding. In contrast, only 13%, 108 out of 820, of the expected nodes were ranked in the top five nodes using a conventional spectral embedding algorithm. The percentage of the top 5 ranked nodes significantly differed across these two embedding methods, *χ*^2^(1, *N* = 1640) = 68.6, *p* < 2.2e-16 (Supplementary Fig. 1).

### Similarity of node representation

**Fig. 3:**
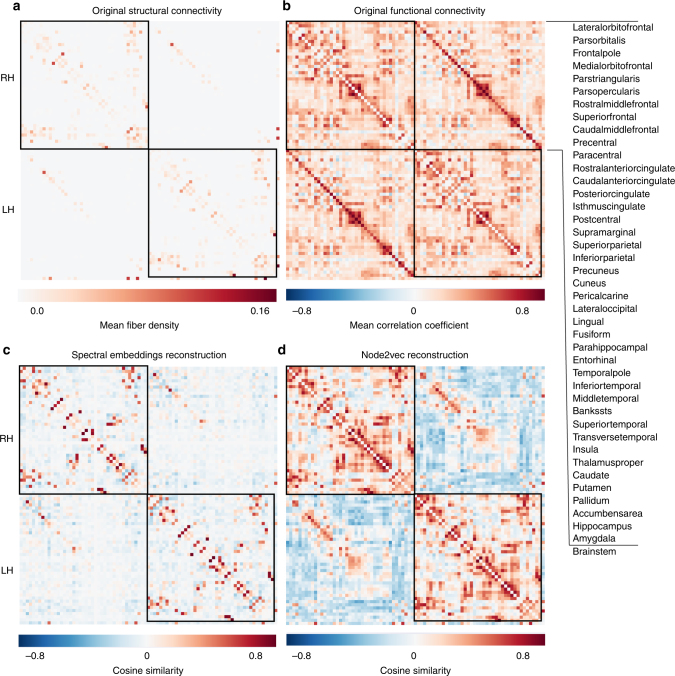
Cosine similarity of the structural connectivity matrix and node embeddings. **a** The original structural connectivity matrix with 83 predefined regions of interest (see Cammoun et al., 2012). Each cell represents a structural connection between a pair of regions. The same regions are used in all matrices. **b** The original mean functional connectivity. **c** Cosine similarity between spectral embeddings. See text for a statistical analysis. **d** Cosine similarity between node2vec embeddings

As implied by the inter-hemispheric analogies test, the relation between the learned CE vectors encompasses meaningful neurobiological information. To further explore this issue and understand the nature of the pair-wise relation between each pair of nodes in relation to functional homotopy, we characterized the similarity between the representations of their respective CE vectors. Specifically, the cosine similarity was calculated between each pair of connectome embedding vectors (Fig. 3). This procedure resulted in a reconstruction of the structural connectivity matrix (embedding reconstruction). We did not expect a perfect reconstruction of the original structural matrix. Rather, we assumed that if CE manages to capture high-level topological attributes, it should be reflected in the CE pair-wise relation.

We first calculated the Spearman’s Rho (rank correlation coefficient) between each embedding reconstruction (node2vec and spectral) and the original matrix of structural connectivity edges that were obtained using diffusion imaging.

Dataset 1: The connectome structural matrix was weakly correlated with the spectral embeddings reconstruction *r*_*s*_ *=* 0.2,* p* *<* 10^−6^, and strongly correlated with the node2vec embeddings reconstruction *r*_*s*_ *=* 0.62,* p* *<* 10^−6^ (Fig. 3). Dataset 2: Similarly to the correlations measured in Dataset 1, the connectome structural matrix was not correlated with the spectral embeddings reconstruction *r*_s_ = −0.02, *p* *=* 0.11 but was strongly correlated with the node2vec embeddings reconstruction *r*_s_ = 0.63, *p* *<* 10^−6^ (Supplementary Fig. 2).

We hypothesized that the difference between the node2vec and the spectral embedding reconstructions may, in part, be due to the tendency of the embedding algorithm to infer missing connections on the basis of existing higher-order relationships. For example, homotopic inter-hemispheric structural connections are not well captured using diffusion imaging^19^. Recently, an analysis which used 32,350 connection reports, expertly collated from published pathway tracing experiments in rats, suggested that about two-thirds of all cortical regions send a homotopic commissural connection^20^. Thus, homotopic inter-hemispheric connections may be better recovered in the node2vec connectivity reconstruction, due to topological features which are captured by the embedding.

To compare the number of homotopic inter-hemispheric connections between the original structural connectome and the reconstructed embedding connectivity matrices, we applied Z-score normalization to each matrix, followed by a threshold applied to the Z-score. Dataset 1***:*** The percentage of homotopic inter-hemispheric edges was 73% for the node2vec reconstructed matrix compared to 48% in the original connectome matrix at 0 threshold. This difference was statistically significant (*χ*^2^ (1, *N* = 82) = 9.76, *p* = 0.001). Similar patterns emerged when various thresholds up to 0.9 were applied, but disappeared at a threshold of 1, with only 44% and 34% of homotopic inter-hemispheric edges apparent for the node2vec matrix and original structural connectivity matrix, respectively. The difference in homotopic inter-hemispheric edges between the spectral embedding reconstruction (53%) and the structural connectivity matrix (48%) was not significant for the 0 threshold (*χ*^2^ (1, *N* = 82) = 0.39, p = .53). Similar results were apparent across thresholds up to 0.9. Dataset 2: Equivalently to the results obtained from Dataset 1, the percentage of homotopic inter-hemispheric edges was 56% for the node2vec reconstructed matrix compared to 21% in the original connectome matrix at 0 threshold. This difference was statistically significant (*χ*^2^ (1, *N* = 82) = 27.9, *p* *<* 10^−6^). Similar patterns emerged when various thresholds up to 0.9 were applied, but disappeared at a threshold of 1, with only 19% and 12% of homotopic inter-hemispheric edges apparent for the node2vec matrix and original structural connectivity matrix, respectively. Note that at 0.6, 0.8 and 0.9 thresholds only non-significant trends were apparent (*χ*^2^ (1, *N* = 82) = 3.12, 3.64 and 3.64, *p* *=* 0.07, 0.056 and 0.056 respectively).

At lower Z-thresholds (0.0–0.1), there were still significant differences in homotopic inter-hemispheric edges between the spectral embedding reconstruction and the structural connectivity matrix (*χ*^2^ (1, *N* = 82) = 6.97 and 6.2, *p* = 0.008 and 0.01). Once increasing the threshold above 0.1 there were no significant differences at any of the thresholds.

### Relation to resting state functional connectivity

As findings reported so far suggest that CE provides a meaningful representation of the structural connectome, we move to examining its relation to functional networks estimated from resting state connectivity. Specifically, statistical dependence among regional time courses is generally called functional connectivity^21^, and numerous previous studies have shown that functional connectivity recorded during long sessions of resting state are robustly related to the underlying structural connectivity^22–26^.

While such resting state connectivity is dependent on a structural backbone, it also expresses higher level interactions between nodes of the network which are not necessarily captured by direct pairwise structural connectivity^24, 27^. For example, in addition to resting-state functional connections between nodes that are directly anatomically connected (direct connections), numerous functional connections also exist between nodes that are not directly anatomically connected (indirect connections), due to indirect interactions throughout the network and the transitivity of cross-correlations^24^. As demonstrated, CE reconstructed matrices contain high-level topological connectivity information. We hypothesized that such information may be associated with resting state functional connectivity to a greater extent than the original structural connectivity matrix, as it may capture a significant proportion of indirect effects.

**Fig. 4:**
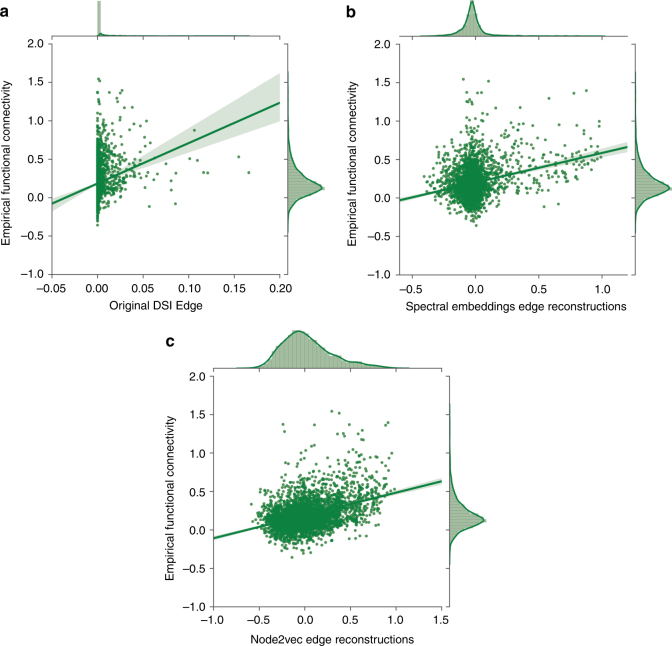
Correspondence between resting state functional connectivity and structural connectivity. Correlation between resting state functional connectivity (after Fisher’s Z-transformation) and **a** original DSI connectivity matrix(*r*_s_ = 0.311, *p* *<* 10^−6^), **b** spectral embeddings matrix reconstructions *(r*_s_ = 0.13, *p* *<* 10^−6^) and **c** node2vec matrix reconstructions (*r*_s_ = 0.328, *p* *<* 10^−6^)

Dataset 1: Indeed, a higher correlation coefficient was obtained between the node2vec reconstructed embedding matrices and the functional connectivity edges (*r*_s_ = 0.328, *p* *<* 10^−6^; Fig. 4c), compared to the correlation between the functional connections and spectral embedding reconstructions edges *(r*_s_ = 0.13, *p* *<* 10^−6^; Fig. 4b), as well as between the functional connections and the original structural edges (*r*_s_ = 0.311, *p* *<* 10^−6^; Fig. 4a). Importantly, when considering node pairs that are not directly connected in the original structural matrix, we obtained a positive correlation between the node2vec reconstruction and the functional connectivity matrix (*r*_s_ = 0.127, *p* *<* 10^−6^) but no significant correlation between the spectral embedding reconstruction and the functional connectivity connections (*r*_s_ = −0.02, *p* = 0.52).

Dataset 2: Similarly to the results obtained with Dataset 1, a higher correlation coefficient was measured between the node2vec reconstructed embedding matrices and the functional connectivity (*r*_s_ = 0.31, *p* *<* 10^−6^; Supplementary Fig. 3c), compared to the correlation between the functional connections and spectral embedding reconstructions (*r*_s_ = 0.15, *p* *<* 10^−6^; Supplementary Fig. 3b), as well as between the functional connections and the original structural edges (*r*_s_ = 0.21, *p* *<* 10^−6^; Supplementary Fig. 3a). When examining the nodes that are indirectly connected in the original structural matrix, we measured a positive correlation between the node2vec reconstruction edges and the functional connectivity matrix *r*_s_ = 0.27, *p* *=* 0.003 but there was no significant correlation between the spectral embedding reconstruction and the functional connectivity connections *r*_s_ = 0.17, *p* *=* 0.069.

These findings suggest that node2vec embeddings capture significant information about functional relations as measured in resting-state functional connectivity.

### Deep learning for structural to functional mapping

To examine whether the mapping between the reconstructed CE and functional connectivity could be further improved, we adopted a supervised deep learning framework. To this end, a representation of edges was required as opposed to the single node embeddings^3^. The mapping between structural embeddings and functional connectivity was learned utilizing a node-pairs representation, while adopting a supervised learning cross-validation scheme (see Methods for details).

**Fig. 5:**
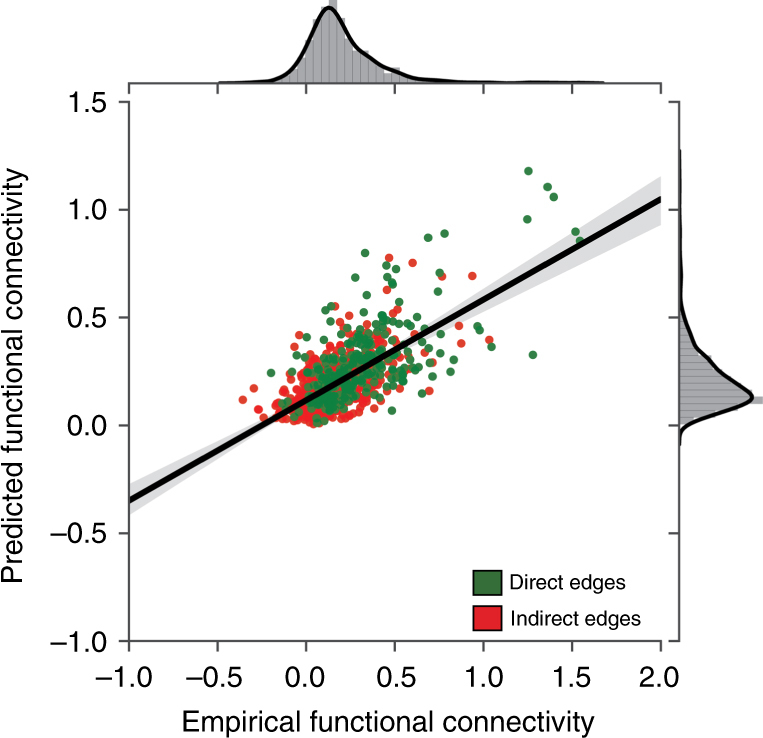
Prediction of resting state functional connectivity from structural embbedings using deep learning. Green and red dots mark direct and indirect edges respectively. A significant correlation between the empirical functional connections and the predicted functional connections is apparent when all connections are taken into account (*r*_s_ = 0.6, *p* *<* 10^−6^), as well within the direct (*r*_s_ = 0.6, *p* *<* 10^−6^) and indirect connections separately (*r*_s_ = 0.52, *p* *<* 10^−6^)

Dataset 1: When assessing the correspondence between predicted functional connections and the empirical functional connections in the testing set, we obtained a strong positive correlation (*r*_s_ = 0.6, *p* *<* 10^−6^) (Fig. 5) which was apparent for both direct connections (*r*_s_ = 0.6, *p* *<* 10^−6^) and indirect connections (*r*_s_ = 0.52, *p* *<* 10^−6^) (respectively green and red symbols in Fig. 5).

Dataset 2: We obtained a strong positive correlation (*r*_s_ = 0.52, *p* *<* 10^−6^) between predicted functional connections and the empirical functional connections in the testing set which was apparent for both direct connections (*r*_s_ = 0.52, *p* *<* 10^−6^) and indirect connections (*r*_s_ = 0.6, *p* *=* 0.001) (respectively green and red symbols in Supplementary Fig. 4).

Thus, the CE encompasses considerable information regarding indirect functional connections matching, or even exceeding prior structure-function correspondences obtained by computer simulation^28^, as well as analytic graph-based models^22^.

As a further validation, we also conducted a simple linear regression analysis. Dataset 1: a positive correlation (*r*_s_ = 0.45, *p* *<* 10^−6^) was obtained between the predicted functional connection and the empirical functional connection in the testing set which was apparent for both direct connections *r*_s_ = 0.41, *p* *<* 10^−6^ and indirect connections (*r*_s_ = 0.32, *p* *<* 10^−6^). Dataset 2: A positive correlation (*r*_s_ = 0.41, *p* *<* 10^−6^) was measured between the predicted functional connection and the empirical functional connection in the testing set which was apparent for both direct connections (*r*_s_ = 0.41, *p* *<* 10^−6^) and indirect connections (*r*_s_ = 0.57, *p* *<* 10^−6^). The fits obtained from the linear regression are below those obtained with our deep learning pipeline.

### Prediction of functional connectivity following FFA lesion

The high predictive power of connectome embeddings provides an opportunity for a new type of predictive model to bridge brain structure and function. One potential application is to predict changes in functional connectivity that result from changes in structure-based connectome embeddings. Specifically, one can create an embedding of the structural connectome after a manipulation such as an artificial lesion or a selective enhancement of specific nodes or edges (e.g., ^29^). This embedding can then be used to predict the functional connectivity following the manipulation.

We utilized the face network as a testbed for exemplifying this framework. Face perception is accomplished via the coordinated activity of a face processing network^30^. One of the major hubs of the face network is the right fusiform face area (FFA)^31, 32^. A lesion in this region may result in acquired prosopagnosia, a severe deficit in face perception^33^. Nevertheless, network-wide effects associated with such a lesion were not yet examined explicitly. However our previous studies revealed that critical regions such as the right FFA serve as a hub in the face network when participants view an intact face but its connectivity is disrupted by physically manipulating the face (e.g., 180 degree rotation of the face^32^). Critically under such disrupted conditions, additional regions (the right LOC, the right IPS and the right inferior temporal cortices) become involved and take on the roles of hubs in this modified network^32^. Similar findings are also obtained when intact faces are perceived by individuals with impaired congenital face processing abilities (congenital prosopagnosia—CP^34^). Hence, we predicted that a lesion to the right FFA would simulate a disruption of the face network that mimics conditions of impaired face perception and would consequently affect the connectivity of the related hubs. Here we attempt to simulate network modifications to the face system that may elicit similar effects to those described above using an artificial lesion.

Employing the CE framework, it is feasible to estimate how a lesion of the right FFA node, a major hub of the face network, would causally affect the entirety of the brain network. A node lesion is performed by setting all of its connections to zero. Using a permutation test with 10,000 iterations (see methods for details), we calculated differences between the pre-lesion and post-lesion simulated functional connectivity. Following the lesion, the functional connectivity of each edge could either decrease (pre-lesion > post-lesion) or increase (post-lesion > pre-lesion). Only simulated edge differences which were greater than all 10,000 permutation differences were considered statistically significant.

**Fig. 6:**
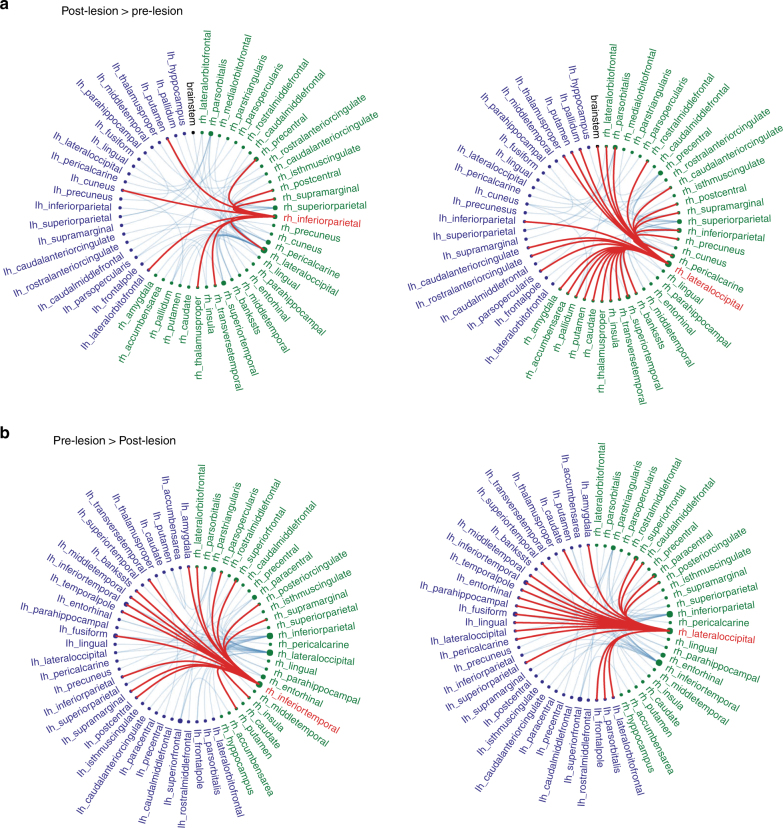
Simulating the effect of an artificial lesion to the right FFA on functional connectivity. Green and purple denote right and left hemispheric nodes respectively and the simulated edge differences which were significantly affected by the lesion are depicted by blue lines (edges) connecting the nodes. Red denotes a selected node and its statistically significant edges. **a** The right LOC and the right IPS nodes had the highest nodal degree in the post-lesion *>* pre-lesion contrast. **b** The right inferior temporal cortex and the right LOC nodes had the highest nodal degree in the pre-lesional > lesion contrast

Dataset 1: The differences between the pre-lesioned and post-lesioned predicted functional connectivity brain network were quantified using a measure of node degree difference, which captures the difference in the number of significant edges attached to a node^5^. Following the lesion, the right lateral occipital cortex (LOC) and the right inferior parietal sulcus (IPS) nodes showed the highest increase in nodal degree (an increase of 27 and 9 edges, respectively). Conversely, the right LOC and right inferior temporal cortex showed the highest decrease in nodal degree as a result of the lesion (an increase of 20 and 17 edges, respectively). Dataset 2: Following the lesion, the right lateral occipital cortex (LOC) and the right inferior temporal cortex nodes showed the highest increase in nodal degree (an increase of 20 and 14 edges, respectively). The inferior parietal cortex was ranked 6^th^ in terms of nodal degree. Conversely, the right LOC and right parahippocampal area showed the highest decrease in nodal degree as a result of the lesion (an increase of 36 and 15 edges, respectively), and the right inferior temporal cortex was only ranked third (10 edges). These simulated results are consistent with the hubs associated with CP but they are also evident when manipulating the network using a behavioral face inversion paradigm^32, 34^(Fig. 6).

Note, that the two datasets produced some minor differences in the ranking of the nodes, which showed the highest increase in nodal degree in both contrasts and in the specific affected edges. Given that the two datasets are completely independent and have distinct preprocessing pipelines, as well as different extracted measures for structural connectivity (See Methods for details), such differences in the observed findings are conceivable.

### Relationship of embeddings and standard topological measures

As is evident, CE captures important topological information. Nevertheless, to investigate whether there is a potential relationship between CE and more standard lower-order topological measures, the correlation between each dimension of the CE and several topological measures was calculated across nodes. Specifically, the Spearman’s correlation coefficient between CE and measures of node centrality (degree, strength, eigenvector centrality), a nodal measure of integration (betweenness centrality) and a nodal measure of segregation (clustering coefficient) was examined.

**Fig. 7:**
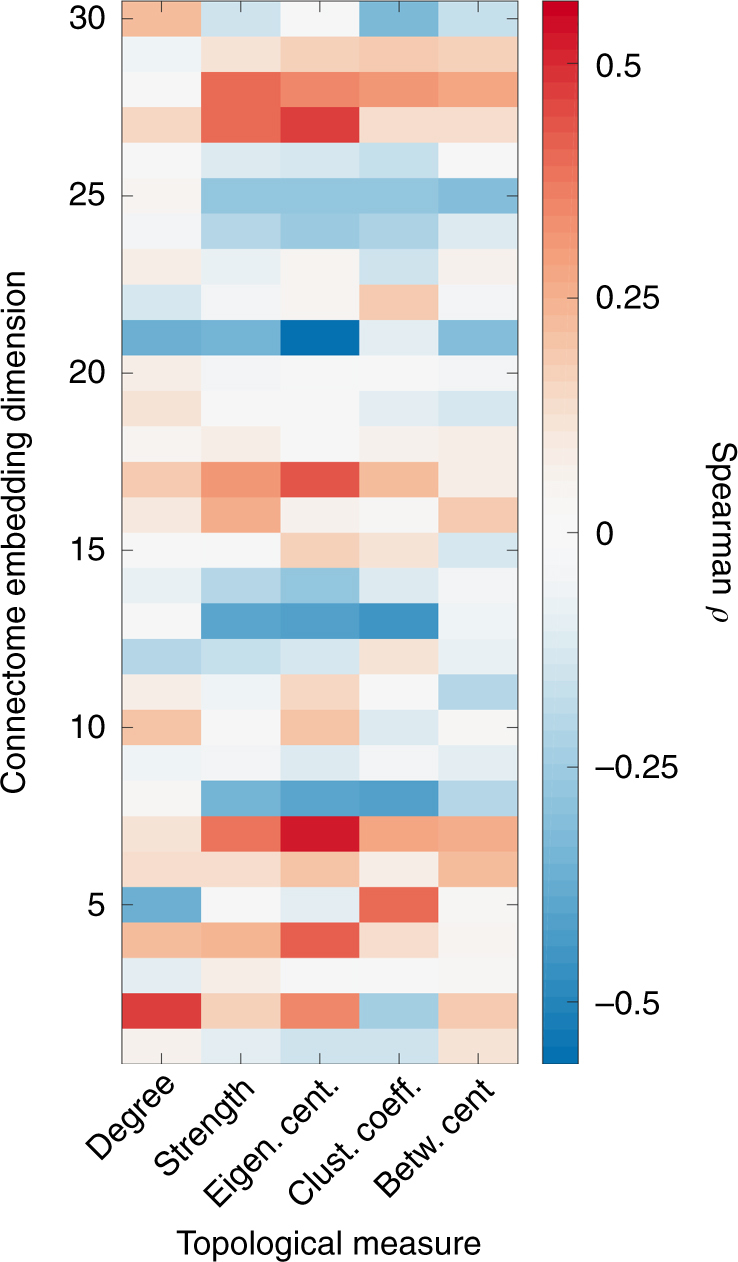
Correlations between CE dimensions and standard topological measures. The eigenvector centrality showed the highest magnitude of correlation to only two of the CE dimensions (maximum Spearman correlation of *ρ* *=* 0.51, *p* < 10^−10^ and minimum correlation of *ρ* *=* −0.57, *p* < 10^−10^). Nevertheless, generally the correlation values between the CE elements and the graph theoretical measures were low and followed no obvious, meaningful pattern

Of the measures tested, the eigenvector centrality showed the highest magnitude of correlation to only 2 of the CE dimensions (maximum Spearman’s correlation of *ρ* *=* 0.51, *p* *<* 10^−6^ and minimum correlation of *ρ* *=* −0.57, *p* *<* 10^−6^). Nevertheless, the correlation values between the CE elements and the graph theoretical measures were low and followed no obvious pattern (Fig. 7). The significant correlations seem sporadic and they do not account for most of the variance associated with CE. This suggests that CE captures network attributes beyond those captured by more standard topological measures.

## Discussion

The utilization of word embeddings techniques such as word2vec for network science in general, and specifically in the context of connectomics, holds great promise^3, 35^. In the current study we demonstrated that CE representation encompasses high-level topological information such as inter-hemispheric similarities. Moreover, CE was able to reveal, at levels superior to previous methodology, the relationship and mutual prediction of functional and structural connectivity, and was able to simulate the effects of localized network lesions on the global pattern of functional connectivity.

The integration of machine learning techniques with models of brain networks is a relatively new domain and examples of successful applications are still limited^35^. Previous studies have mostly utilized embeddings as a dimensionality reduction step of fMRI data for subsequent machine learning tasks such as classification of patients with schizophrenia^36^, depression^37^, Alzheimer’s disease^38^ and multiple sclerosis^39^. Differences between structural connectomes and deterioration of connectomes as a result of edge deletion have previously been investigated using the average similarity of heat diffusion^40^. However, the word2vec family of models together with deep learning algorithms have not yet been applied in the context of brain networks. Furthermore, this study is the first to create a comprehensive machine-learning framework which translates meaningful structural embeddings to functional connectivity, giving rise to a novel predictive model of how functional connectivity is affected by alterations of structural elements which may become useful in the investigation of abnormal brain networks.

To test whether CE vector representations reflect known attributes of brain topology/topography and can be interpreted and manipulated using linear operations, several benchmarks were tested on two independent datasets. Critically, we performed an extensive validation with a rigorously preprocessed subset of the Human Connectome Project (100 subjects) and replicated the main results. Note that due to technical differences in data acquisition, the preprocessing pipeline, as well as the extracted measure for structural connectivity were different between the datasets which further strengthens the generalizability of our approach and methods. The initial inter-hemispheric analogies test demonstrated that CE vector representations capture the known functional homotopy organizational principle^11–14^. As predicted, the relation between most pairs of regions in one hemisphere was analogous to the same pairwise relation in the other hemisphere, with the CE approach exhibiting superior performance over previous embedding techniques.

Next, we examined whether the correlation between CE and the structural connectivity matrix reflects higher-order attributes over and above the dyadic pattern of structural connections. This indeed turned out to be the case as homotopic inter-hemispheric connections were more prominent in the CE connectivity reconstruction compared with the original structural connectivity matrix, due to topological features which are captured by the embedding such as homotopic inter-hemispheric connectivity. Moreover, the CE matrices were more strongly correlated with resting state functional connectivity than the original connectivity matrix. Furthermore, a deep learning algorithm was utilized to improve the mapping between CE and functional connectivity utilizing CE representations. This mapping produced high correlation coefficients between the predicted and empirical functional connectivity values, both for direct, as well as in-direct connections, which are more difficult to estimate^41^. The CE approach outperformed previous models of structure-function correspondences^22, 28^. Future studies may utilize the same predictive algorithm to predict missing structural connectivity in species and modalities where only partial structural connectivity data is available^42^.

To capitalize on the high predictive power of CE-functional mapping, we tested whether it is possible to predict changes in functional connectivity that result from changes in structure-based connectome embeddings. Specifically, we used the face network as a test-bed and simulated a structural lesion to the right FFA, a well-documented hub of the face network^31, 32^. The results of the simulation aligned well with empirical findings exploring the face network. Hyper-connectivity in the right LOC, inferior temporal cortex and IPS which was reported in previous empirical studies^32, 34, 43^, was also predicted by our CE-based model. In line with previous work^44, 45^, our findings suggest that network-wide functional changes can result from a localized manipulation such as the suppression of a single node.

Our results, along with the modeling framework, make a further step towards the possibility of examining causality in the context of structural and functional network alterations. The same framework can be used to induce simulated lesions, overexpression of nodes, edges, as well as entire sub-networks. Such simulations might help to elucidate the structural basis for network alterations which occur in neuro-developmental disorders such as Autism Spectrum Disorder (ASD) in which hyper-connectivity is apparent^29^, and developmental dyslexia and acquired prosopagnosia where the left and right fusiform gyri are focally implicated, respectively^31, 46^. Moreover, one may simulate the changes in network topology observed in normal participants under different cognitive and perceptual demands. For example, a number of studies have demonstrated the complementary extrinsic and intrinsic networks associated with external inputs and intrinsically driven processing respectively.^47^.

The embedding algorithm (node2vec^3^) and the parameters employed in the current study are not necessarily optimal, and are subject to further improvement in future extensions of this work. Our work suggests that CE provides a powerful approach for exploring the higher-order network structure of connectome data sets, with potential applications in modeling and comparing individual differences in human connectomes across development and in clinical conditions. Another future application is to use CE to uncover relationships and homologies among brain architectures across species.

## Methods

### MRI Data

Datasets: The analyses were conducted on a dataset of 40 subjects (Dataset 1) and validated on an independent dataset of 100 subjects (Dataset 2).

Dataset 1 (Lausanne). Prior to collection of MRI data, the project was submitted for approval to the University of Lausanne Ethics Committee [Institutional Review Board (IRB)]. The study protocol was approved by the local IRB and informed written consent from each subject was obtained prior to study inclusion.

Forty healthy subjects (16 females; 25.3 ± 4.9 y old), with no relevant medical or psychiatric history, underwent an MRI session on a 3 T Siemens Trio scanner with a 32-channel head coil (previously reported on^22, 48^). T1 weighted magnetization-prepared rapid acquisition with gradient echo (MPRAGE) sequence was 1-mm in-plane resolution and 1.2-mm slice thickness. Diffusion Spectrum Imaging (DSI)^49^ included 128 diffusion weighted volumes + 1 reference b0 volume, maximum b-value 8000 s/mm2, 2.2 × 2.2 × 3.0 mm voxel size and with TR 6800 ms and TE 144 ms. BOLD contrast was recorded with a gradient echo EPI sequence of 3.3-mm in-plane resolution and 3.3-mm slice thickness and with TR 1920 ms and TE 30 ms.. DSI, resting-state fMRI, and MPRAGE data were processed using the Connectome Mapping Toolkit^50^

Segmentation of gray and white matter was based on MPRAGE volumes. Cerebral cortex was parcellated into a set of 83 regions of the Desikan-Kiliany atlas^51, 52^. Whole-brain streamline tractography was performed on reconstructed DSI data^53^. During the resting-state fMRI acquisition, subjects were lying in the scanner with eyes open for 9 min. Functional data preprocessing included motion correction, white matter, cerebrospinal fluid, global and movement signals regression, linear detrending, motion scrubbing, and low-pass filtering^54, 55^. Average time series were computed for each cortical region and functional connectivity was estimated as Pearson cross-correlation^22^.

Dataset 2 (Human Connectome Project; HCP). Prior to collection of MRI data, the HCP scanning protocol was approved by the local Institutional Review Board at Washington University in St. Louis and informed written consent from each subject was obtained prior to study inclusion. Full details on the HCP dataset have been published previously^56–58^. Out of the HCP 900 subjects data release, 100 hundred unrelated subjects were used for the analysis^58^. Individual structural and functional connectomes were estimated following the same processing procedures as detailed in Amico & Goni^59^.

Structural data: Very high-resolution acquisitions (1.25 mm isotropic) were obtained by using a Stejskal–Tanner (monopolar) diffusion-encoding scheme. Sampling in q-space was performed by including 3 shells at *b* = 1000, 2000, and 3000 s/mm2. For each shell corresponding to 90 diffusion gradient directions and 5 *b* = 0’s acquired twice were obtained, with the phase encoding direction reversed for each pair (i.e., LR and RL pairs). Directions were optimized within and across shells (i.e., staggered) to maximize angular coverage using the approach of Caruyer, et al. (2011)^60^ (http://www-sop.inria.fr/members/Emmanuel.Caruyer/q-space-sampling.php), and form a total of 270 non-collinear directions for each PE direction. Correction for EPI and eddy-current-induced distortions in the diffusion data was based on manipulation of the acquisitions so that a given distortion manifests itself differently in different images^61^. To ensure better correspondence between the phase-encoding reversed pairs, the whole set of diffusion-weighted (DW) volumes is acquired in six separate series. These series were grouped into three pairs, and within each pair the two series contained the same DW directions but with reversed phase-encoding (i.e., a series of Mi DW volumes with RL phase-encoding is followed by a series of Mi volumes with LR phase-encoding, *i* = [1–3]).

The HCP DWI data were processed following the MRtrix3^62^ guidelines (http://mrtrix.readthedocs.io/en/latest/tutorials/hcp_connectome.html). In summary, a tissue-segmented image appropriate for anatomically constrained tractography was generated (ACT^63^, MRtrix command 5ttgen); the multi-shell multi-tissue response function was estimated (ref. ^64^, MRtrix command dwi2response msmt_5tt) and a multi-shell, multi-tissue constrained spherical deconvolution was performed (ref. ^65^, MRtrix dwi2fod msmt_csd); afterwards, an initial tractogram was generated (MRtrix command tckgen, 10 million streamlines, maximum tract length = 250 millimeters, FA cutoff = 0.06) and the successor of spherical-deconvolution Informed Filtering of Tractograms (SIFT2,^66^) methodology (MRtrix command tcksift2) was applied. Both SIFT^67^ and SIFT2^66^ methods provides more biologically meaningful estimates of structural connection density^66^. Finally, the SIFT2 outputed streamlines were parcellated into a set of 82 regions of the Desikan-Kiliany atlas^51, 52^ (MRtrix command tck2connectome).

Functional data: The fMRI resting-state runs (HCP filenames: rfMRI_REST1 and rfMRI_REST2) were acquired in separate sessions on two different days, with two different acquisitions (left to right or LR and right to left or RL) per day. For all sessions, data from both the left-right (LR) and right-left (RL) phase-encoding runs were used to calculate connectivity matrices^56, 57^.

The data was processed using the HCP functional pipeline^56, 57^. This pipeline included artifact removal, motion correction and registration to standard space. Full details on the pipeline can be found in^56, 57^. The main steps were: spatial (minimal) pre-processing, in both volumetric and grayordinate forms (i.e., where brain locations are stored as surface vertices^57^; weak highpass temporal filtering (>2000s full width at half maximum) applied to both forms, achieving slow drift removal. MELODIC ICA^68^ applied to volumetric data; artifact components identified using FIX^69^. Artifacts and motion-related time courses were regressed out (i.e., the 6 rigid-body parameter time-series, their backwards-looking temporal derivatives, plus all 12 resulting regressors squared) of both volumetric and grayordinate data^57^. Furthermore, global gray matter signal was regressed out of the voxel time courses^70^; a bandpass first-order Butterworth filter in forward and reverse directions [0.001 Hz, 0.08 Hz]^70, 71^ was applied (Matlab functions butter and filtfilt); the voxel time courses were Z-scored and then averaged per brain region of the 82 regions of the Desikan-Kiliany atlas^51, 52^, excluding outlier time points outside of 3 standard deviation from the mean, using the workbench software^72^ (workbench command-cifti-parcellate).

Pearson correlation coefficients between pairs of nodal time courses were calculated (MATLAB command corr), resulting in a symmetric connectivity matrix for each fMRI session of each subject. Finally, the mean connectivity matrix was calculated for each subject across all 4 acquisitions.

For both of the datasets, the subsequent structural connectivity analyses and modeling were carried out on a group consensus matrix, built by averaging over all existing connections (expressed as fiber densities) that were present in at least 25% of participants^48^. For the functional data, the consensus matrix was built from averaging over all participants.

### Word and network embedding

Word2vec mainly consists of two models; skip-gram and the continuous bag of words (CBOW). Briefly, given a corpus of words *w* and their context *c*, the goal is to maximize the conditional probability *p*(*c*|*w*; *θ*) in the skip-gram model or *p*(*w*|*c*; *θ*) in the CBOW model by estimating the parameters *θ*^73^. The produced parameters or vectors capture linguistic regularities and may be the basis for various subsequent tasks^7, 8^.

The analog for context in the network domain are streams of randomly generated walks in the network. The way the network sentences are generated distinguishes between different approaches of network embeddings. One of the recent implementation of network node embeddings is node2vec which controls for the depth of the random walk using 2 parameters allowing for local or global random walks which lead to different representation of the nodes (Fig. 1a-e)^3^. Compared with unsupervised feature learning approaches, which utilize the spectral properties of graphs, the node2vec model has been shown to have higher predictive power across a range of subsequent supervised learning node classification tasks and link prediction of edges^3^. Moreover, it has been shown that similar network embeddings algorithms capture the k-step (*k* = 1, 2, 3,..) relation between each vertex and its k-step neighbors in the graph while projecting all such k-step relational information into a common subspace^10^.

We used Node2vec and underlying Gensim python package^3, 74^ to run the CBOW node2vec algorithm 500 times on the structural connectivity matrix, as it can produce different outcomes in each iteration. Each iteration consisted of 800 random walks with a length of 20 steps. The dimension of the embedded vectors was set to 30 (the length of each vector which represents a node) and the window size (the number of steps from each node) determining the context of each node, was set to 3. Parameters of the algorithm were set to correspond to a localized random walk (*p* = 0.1, *q* = 1.6). The walk probabilities were weighted according to the weight of the connectome edges.

### Deep learning better predicts functional connectivity

Previously, Grover and Leskovec (2016) have shown that the Hadamard operation, the element-wise multiplication between pair of vectors, was efficient for learning edge features across various domains. The mapping between structural embedding and functional connectivity can be learned in such a setting utilizing node-pairs representations, while adopting a supervised learning cross validation scheme. Specifically, the edges of the mean functional connectivity matrix were randomly divided into training (75%) and testing sets (25%). A deep learning multi-layer perceptron model, as implemented in Keras with a Tensorflow^75^, was used in which the independent variables were defined as the Hadamard embbedings and the dependent variable was defined as the functional connectivity. We first optimized the architecture of the network using a cross validation grid-search over the parameter space using only the training set. This yielded a 4 layer fully connected network with 350 neurons in each layer, dropout rate of 0.1, rectified linear units (RELU) activation function, batch size of 140, 170 epochs and Adam optimizer.

### Prediction of functional outcome due to lesion

To test the effect of an artificial lesion on the functional connectivity, we first constructed new 500 connectome embbedings following a right FFA removal (post-lesion embeddings compared with the original pre-lesion embeddings) as this is a major hub of the face network^31, 32^. This was done by running the node2vec algorithm 500 times with random initializations on the structural connectivity matrix after setting to 0 all of the right FFA edges. As the embedded vector elements may change due to the random weights initialization conditions (but the cosine similarity between vectors is stable), the learned mapping between the pre-lesioned embedding and the functional connectivity mapping is not generalizable to embeddings of another connectome. Therefore, we devised a training procedure whose goal is to learn a mapping between the connectome embeddings to the functional connectivity that is invariant to the weight initialization conditions. To this end, we implemented a nested cross validation scheme in which the mapping between pre-lesion structural embeddings and functional connectivity is trained across many random weights initializations of the connectome embeddings using only a subset of the edges and connectome embeddings to avoid overfitting the data. Specifically, the edges are divided into 3 folds (sub groups) such that in each iteration, 2 folds are used for training (2268 edges) and 1 fold is used for testing (1134 edges). Moreover, the 500 connectome embeddings are also split randomly 3 times with a training set of 90% of the connectome embeddings (450 out of 500), and a testing set of 10% (50 out of 500). The predictions were always made on embedding vectors and edges which were not in the training set. Furthermore, the training is conducted on the pre-lesion connectome embeddings and the prediction is applied to the pre-lesion, as well as to the post-lesion connectome embeddings.

Finally, a permutation resampling test was performed with 10,000 iterations to compare each edge between the pre-lesion and the post-lesion connectome predictions. Specifically, in each iteration the groups are randomly permuted and the difference of each edge is calculated between the two groups, effectively forming the null hypothesis that the groups are invariant under label permutation. Only edges which had a mean difference greater than the permuted mean difference (either for pre-lesion > post-lesion or post-lesion > pre-lesion) across all 10,000 iterations were considered significantly different.

### Code availability

Code was written using standard python functions and freely available packages (see connectome embedding implementation at https://github.com/gidonro/Connectome-embeddings). The original node2vec implementation can be found at https://github.com/aditya-grover/node2vec.

### Data availability

Human Connectome dataset: Data were provided by the Human Connectome Project, WU-Minn Consortium (Principal Investigators: David Van Essen and Kamil Ugurbil; 1U54MH091657) funded by the 16 NIH Institutes and Centers that support the NIH Blueprint for Neuroscience Research; and by the McDonnell Center for Systems Neuroscience at Washington University (10.1038/nn.4361).

Lausanne dataset: The relevant data are available from the authors upon reasonable request.

## Electronic supplementary material

Supplementary Information
