# Supplementary material for: Mapping higher-order relations between brain structure and function with embedded vector representations of connectomes
Source: Nat Commun. 2018 Jun 5;9:2178. doi: 10.1038/s41467-018-04614-w (PMC5988787; doi:10.1038/s41467-018-04614-w)
Supplement: Supplementary file 1 — Supplementary Information [file 41467_2018_4614_MOESM1_ESM.pdf]

Mapping higher-order relations between brain structure and function with embedded vector representations of connectomes

Supplementary Information

Rosenthal et al.

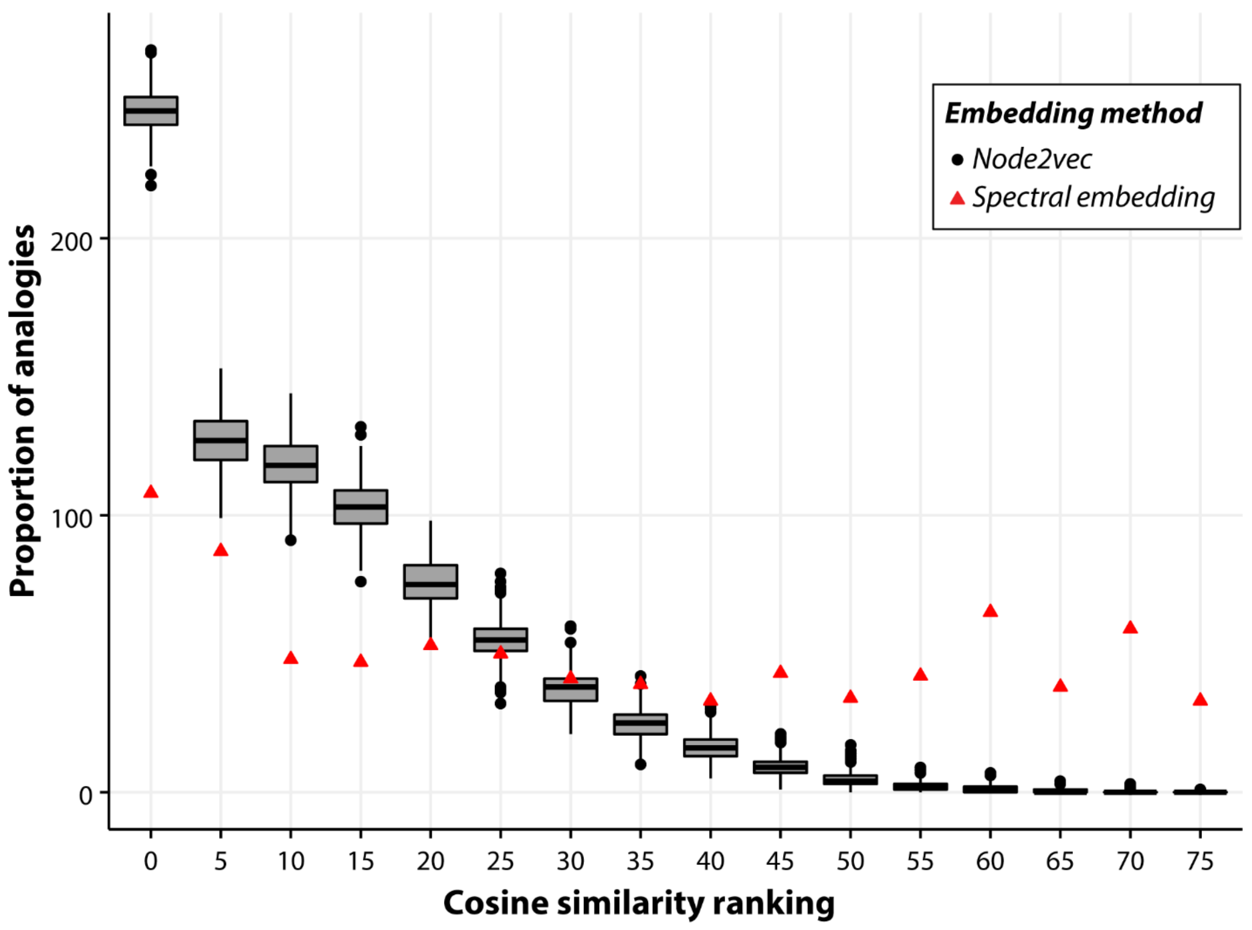

Supplementary Figure 1 – Performance of two node embedding algorithms on the inter-hemispheric analogies test

The inter-hemispheric analogies test evaluated the capacity of two node embeddings to infer the relation between each pair of nodes in one hemisphere, given the same pairwise relation in the other hemisphere. Predictions across all pairwise analogies were ranked, such that a lower rank corresponds to better performance. Across 500 iterations of the node2vec algorithm, the ranking of the expected nodes were binned into bins of 5. The boxplots represent the binning of the ranking across 500 node2vec permutations. The band inside the box represents the median, the lower and upper hinges correspond to the first and third quartiles (the 25th and 75th percentiles) and the whiskers represent 1.5 times the inter-quartile range (the distance between the first and third quartiles). The red triangles represent the binning of the spectral ranking.

Importantly, the node2vec algorithm produced a higher proportion of expected nodes in the lowest rank bin (0-5 ranking). Note the relatively high proportion of spectral embedding analogies with high ranking which suggest worst performance in this task.

This result demonstrates that node2vec vector embeddings successfully encompass functional homotopy information.

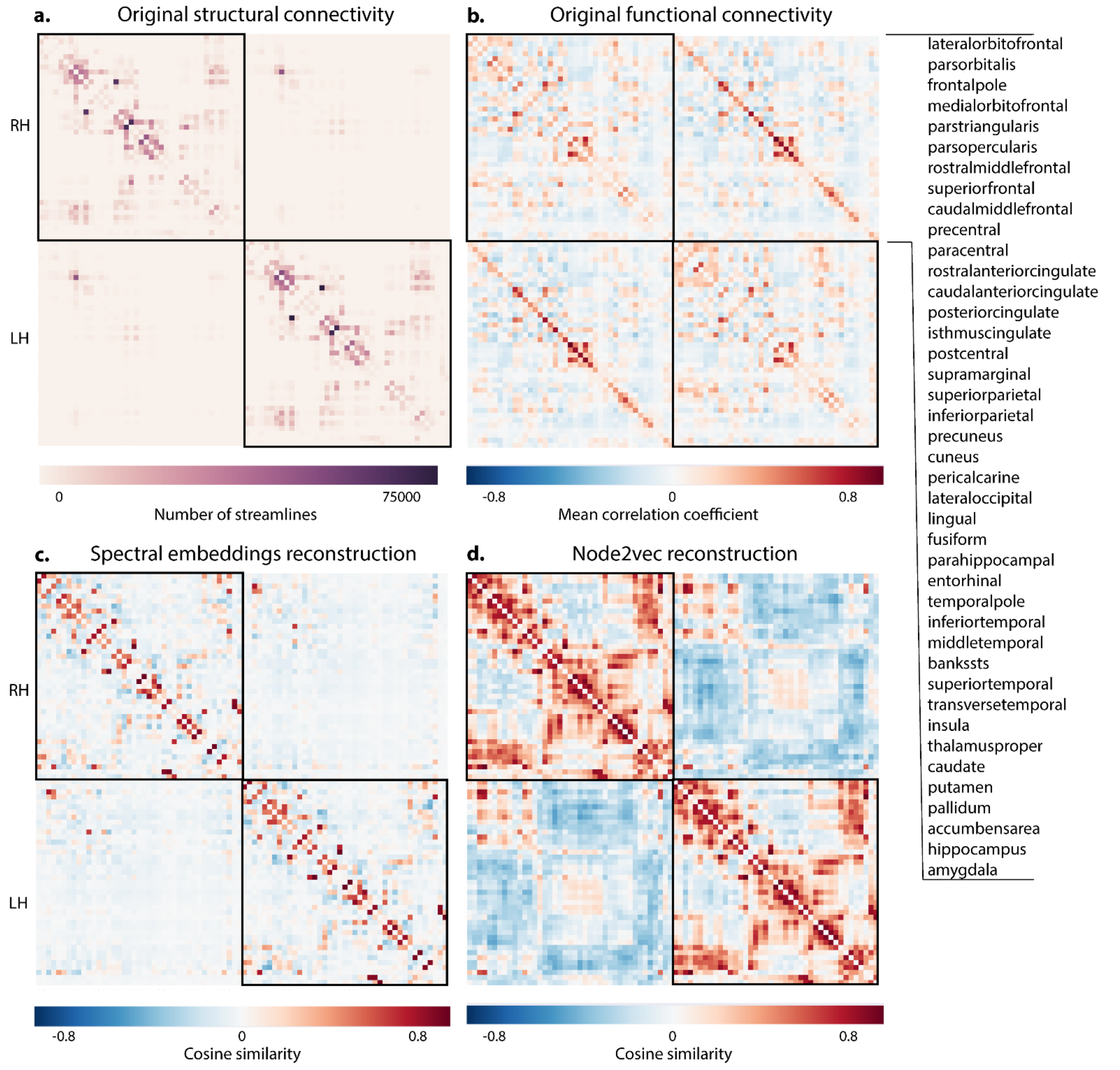

Supplementary Figure 2 – Cosine similarity of the structural connectivity matrix and node embeddings.

a. The original structural connectivity matrix with 82 predefined regions of interest (see Cammoun et al., 2012). Each cell represents a structural connection between a pair of regions. The same regions are used in all matrices. **b**. The original mean functional connectivity **c.** Cosine similarity between spectral embeddings. Note the visual similarity between this matrix and the original DSI matrix. See text for a statistical analysis. **d.** Cosine similarity between node2vec embeddings. Note that the matrix is much denser compared to the original DSI matrix

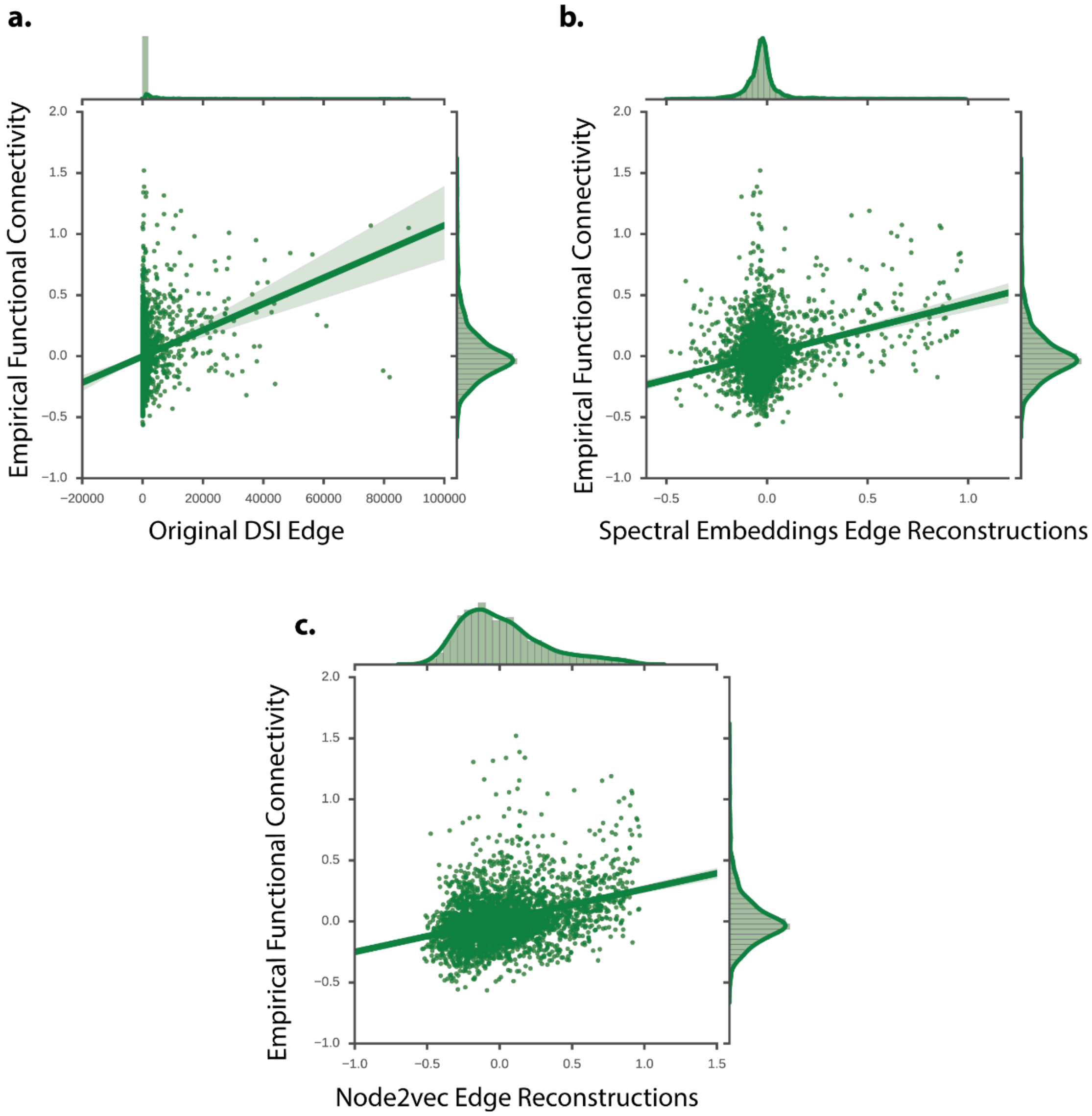

Supplementary Figure 3 – Correspondence between resting state functional connectivity and structural connectivity.

Correlation between resting state functional connectivity (after Fisher's z transformation) and ***a.*** original DSI connectivity matrix ($r_s = 0.21$, $p < 10^{-6}$) ***b.*** Spectral embeddings matrix reconstructions ($r_s = 0.15$, $p < 10^{-6}$) and ***c.*** node2vec matrix reconstructions ($r_s = 0.31$, $p < 10^{-6}$)

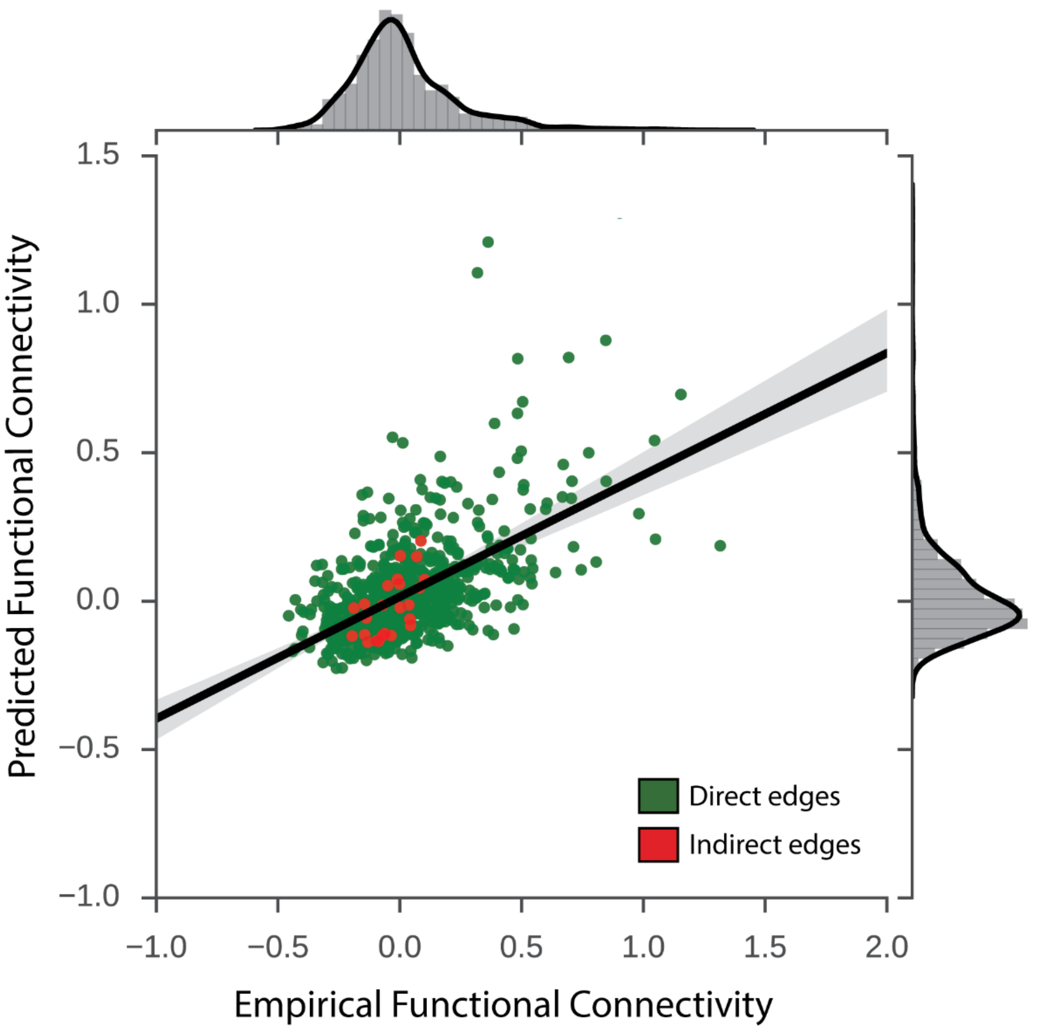

Supplementary Figure 4 Prediction of resting state functional connectivity from structural embbedings using deep learning

Green and red dots mark direct and indirect edges respectively. A significant correlation between the empirical functional connections and the predicted functional connections is apparent when all connections are taken into account ($r_s = 0.52$, $p < 10^{-6}$), as well within the direct ($r_s = 0.52$, $p < 10^{-6}$) and indirect connections separately *(*$r_s = 0.6$, $p = 0.001$).

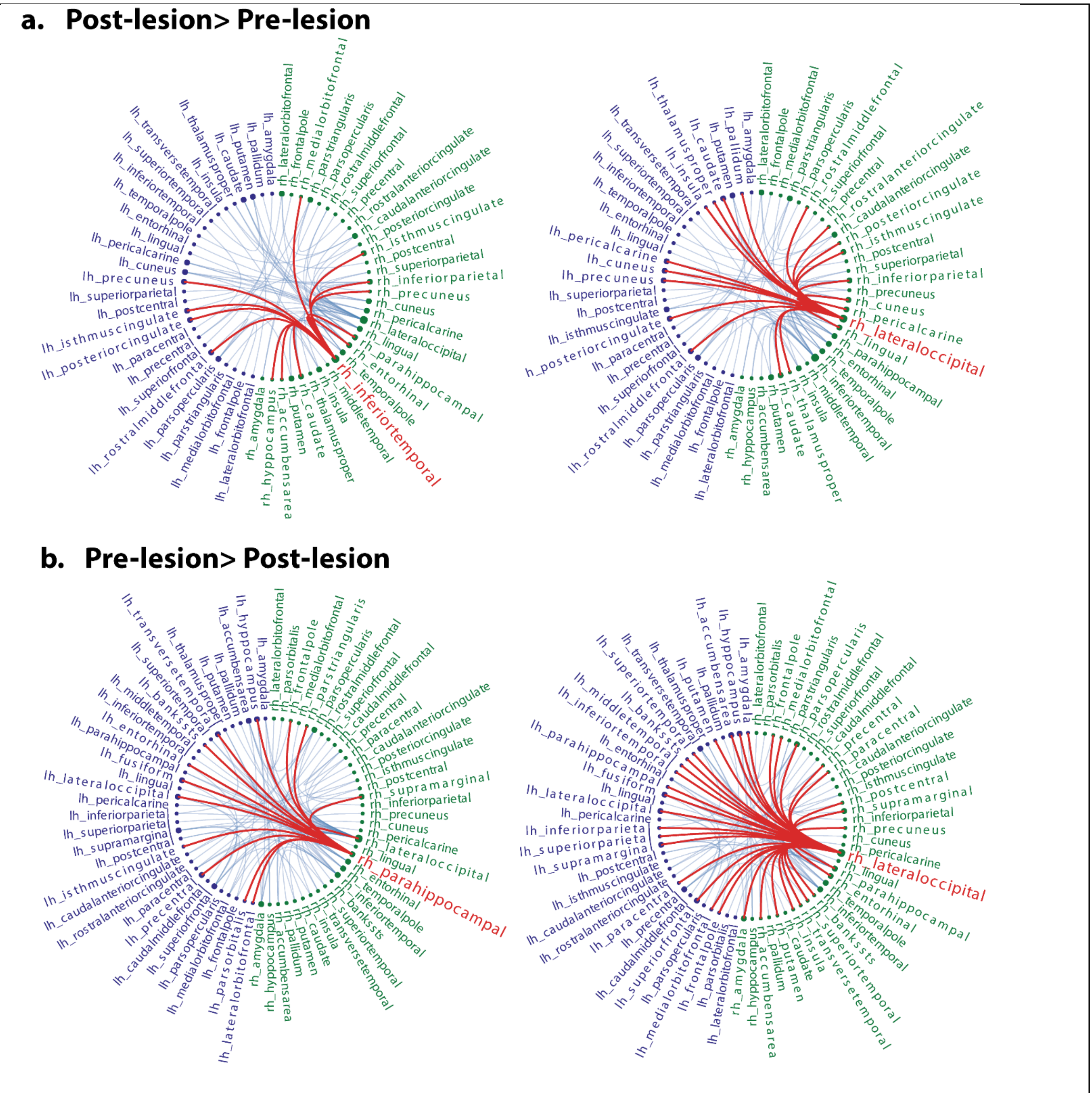

Supplementary Figure 5 Simulating the effect of an artificial lesion to the right FFA on functional connectivity

Green and purple denote right and left hemispheric nodes respectively and the simulated edge differences which were significantly affected by the lesion are depicted by blue lines (edges) connecting the nodes. Red denotes a selected node and its statistically significant edges.

a. The right LOC and the right inferior temporal cortex nodes had the highest nodal degree in the post-lesion > pre-lesion contrast

b. The right LOC and the right parahippocampal area nodes had the highest nodal degree in the pre-lesional > lesion contrast
